# 

**Published:** 1867-11

**Authors:** 


### CONTENTS.

ORIGINAL COMMUNICATIONS.

Dental Society of Western New York ..... 457
Certain effects of Malaria ..... 475

PROCEEDINGS OF SOCIETIES.

The Western New York Dental Association ..... 484
Organization of the Tennessee Dental Association ..... 485

EDITORIAL.

Legal Recognition and protection ..... 489
The Dental Association of Western New York ..... 492
Replanted Teeth ..... 493
Inherited Diseases ..... 494
Exostosis ..... 495
Transactions of the American Dental Association, 1867 ..... 497
Personal ..... 497
Financial ..... 498
The Lake Erie Dental Association ..... 498
Dental meeting ..... 498

